# Supplementary material for: Social, psychological and health characteristics associated with stability and change in adult alcohol consumption
Source: PLoS One. 2022 Nov 11;17(11):e0277511. doi: 10.1371/journal.pone.0277511 (PMC9651548; doi:10.1371/journal.pone.0277511)
Supplement: Attachment — Submitted filename: Response to Reviewers.docx [file pone.0277511.s002.docx]

**PONE-D-22-04437R1**

**Social, psychological and health characteristics associated with stability and change in adult alcohol consumption**

**PLOS ONE

Author response to journal requirements and reviews**

Journal Requirements:

To our knowledge we have not cited retracted papers

Reviewer 2:

*I found the authors responses to the requirements of the journal adequate. I appreciate the authors’ response to the issue of studying sex differences raised by Reviewer 1.*

Response: we appreciate the reviewer’s positive evaluation of our response to the previous reviews.

*With regard to the lack of hypotheses, can the authors briefly state somewhere in the manuscript that these analyses were exploratory?*

Response: Thank you for this suggestion. We have stressed the explorative nature of the study in both the introduction and the conclusion.

Line 106: The aims of this explorative study are to describe the prevalence of different adult-life alcohol consumption trajectories among Danish men and to analyze social, psychological, lifestyle and health characteristics associated with these adult-life consumption trajectories.

Line 539: The findings of this explorative study suggest that the majority of Danish men drink moderately in the life period from young adulthood to late midlife, and in a statistical sense stable moderate consumption is “normal” in Denmark.

*I agree with R1 on point 13. The sentence “…participants who were abstaining from alcohol had the lowest intelligence score…” could be reworded to just state “…participants who were abstaining from alcohol had an intelligence score of M = 95.81…” to avoid any confusion. While the readership of PLOS One might not misinterpret this sentence, I think erring on the side of caution can’t hurt. This is a sensitive topic and if there is a chance that readers could infer that abstainers have an IQ below the normal range (even if the authors do not imply this direct relationship), that could be problematic.*

Response: We have followed the reviewer’s suggestion in the revised manuscript:

Line 297: Table 2 shows young adult characteristics in relation to consumption trajectories. The highest intelligence score and educational level were observed among moderate drinkers, while participants who were abstaining from alcohol had a mean intelligence score of 95.81.

*I found the response to my concern about the methods section appropriate, especially the reference to the meta-analyses of alcohol intake in adolescents. I also found the response to my concern about the 1-21 drinks per week categorization acceptable given Danish standards for sensible drinking and few differences between subgroups who drink 1-9 vs. 10-21. In general, all of my concerns were addressed.*

Response: Thanks again for the positive comments.

*A minor note: In response to R1’s request for more info on exclusion criteria, the authors mention “mental retardation” which I believe is now called “intellectual disability.”*

Response: This is correct and line 156 has been revised accordingly: All Danish men – except those with disqualifying disease such as intellectual disability, diabetes, and epilepsy – must appear before the draft board at age 18 or seek permission to appear later.
